# Perforin deficiency attenuates collagen-induced arthritis

**DOI:** 10.1186/ar1758

**Published:** 2005-05-20

**Authors:** Kristin Bauer, Annika Knipper, Hoang Tu-Rapp, Dirk Koczan, Hans-Jürgen Kreutzer, Horst Nizze, Eilhard Mix, Hans-Juergen Thiesen, Rikard Holmdahl, Saleh M Ibrahim

**Affiliations:** 1 Institute of Immunology, University of Rostock, Rostock, Germany; 2 Institute of Pathology, University of Rostock, Rostock, Germany; 3 Institute of Neurology, University of Rostock, Rostock, Germany; 4 Section for Medical Inflammation Research, Lund University, Lund, Sweden

## Abstract

Collagen-induced arthritis (CIA), an approved animal model for rheumatoid arthritis, is thought to be a T cell-dependent disease. There is evidence that CD8^+ ^T cells are a major subset controlling the pathogenesis of CIA. They probably contribute to certain features of disease, namely tissue destruction and synovial hyperplasia. In this study we examined the role of perforin (pfp), a key molecule of the cytotoxic death pathway that is expressed mainly in CD8^+ ^T cells, for the pathogenesis of CIA. We generated DBA/1J mice suffering from mutations of the pfp molecule, DBA/1J-pfp^-/-^, and studied their susceptibility to arthritis. As a result, pfp-deficient mice showed a reduced incidence (DBA/1J-pfp^+/+^, 64%; DBA/1J-pfp^-/-^, 54%), a slightly delayed onset (onset of disease: DBA/1J-pfp^+/+^, 53 ± 3.6; DBA/1J-pfp^-/-^, 59 ± 4.9 (mean ± SEM), and milder form of the disease (maximum disease score: DBA/1J-pfp^+/+^, 7.3 ± 1.1; DBA/1J-pfp^-/-^, 3.4 ± 1.4 (mean ± SEM); *P *< 0.05). Concomitantly, peripheral T cell proliferation in response to the specific antigen bovine collagen II was increased in pfp^-/- ^mice compared with pfp^+/+ ^mice, arguing for an impaired killing of autoreactive T cells caused by pfp deficiency. Thus, pfp-mediated cytotoxicity is involved in the initiation of tissue damage in arthritis, but pfp-independent cytotoxic death pathways might also contribute to CIA.

## Introduction

Collagen-induced arthritis (CIA) is an experimental model of arthritis inducible in susceptible strains of mice, for example DBA1/J, by immunization with bovine collagen type II (CII) in complete Freund's adjuvant (CFA) [1-4]. The development of CIA is known to depend on T cells, and disease susceptibility is linked to the major histocompatibility complex (MHC) region [5]. After T cell activation an inflammatory cascade involving T cells, macrophages/monocytes, B cells, and activated synoviocytes, is triggered. Different types of leucocytes and synovial cells produce a complex array of cytokines and other soluble mediators, which are thought to be responsible for cartilage destruction and bone erosion [6-9]. Some of the main features of disease are synovial hyperplasia and mononuclear cell infiltration. Factors contributing to this phenomenon are unknown; however, an imbalance between rates of cell proliferation and cell death (apoptosis) has been suggested by recent studies of rheumatoid synovium demonstrating that apoptosis of synovial cells and infiltrating lymphocytes was common *in situ *[10,11]. In the immune system, apoptosis is involved in development and in the negative selection of lymphocytes. It is also crucial in downregulating immune responses to foreign antigens. Cytotoxic T lymphocytes and other killer cells can eliminate their targets by the induction of cell death. All of these functions are primarily mediated through the receptors Fas/APO-1, tumor necrosis factor receptor 1 (TNFR1; p55 TNFR) and the perforin (pfp)/granzyme pathway [12,13].

Perforin is expressed mainly in activated cytotoxic T lymphocytes (CTLs) and natural killer (NK) cells, although some reports suggest its expression in microglia as well [14]. In CTLs, pfp is stored in cytoplasmic granules and is a major effector of cytolysis by these cells. On pfp release it inserts itself into the plasma membrane of target cells and polymerizes into pore forming aggregates. Pores of pfp lead to osmotic lysis of target cells and induce apoptosis by allowing granzymes to enter the target cells. Perforin-deficient mice have confirmed its function as an effector molecule and in the immune response to viruses and tumors as well as in other aspects of immune regulation such as activation-induced cell death (AICD), antibody production and spontaneous autoimmunity [15-17]. NOD mice, an animal model of insulin-dependent diabetes mellitus with a mutation of the pfp gene (NOD/pfp-mice), develop diabetes with highly reduced incidence and markedly delayed onset, pointing to a role of the pfp death pathway in tissue damage in this disease [18].

The role of pfp in arthritis is not clear, although some observations suggest a role in disease pathogenesis, for example pfp-expressing CTLs has been demonstrated in the rheumatoid synovium, and CD8-deficient mice seem to be less susceptible to induction of collagen-induced arthritis [19].

It is conceivable that the pfp/granzyme pathway could contribute to the pathology of arthritis in at least two ways: promotion of autoimmunity by blocking peripheral tolerance and AICD or destruction of target tissues. In this study we attempted to evaluate the role of the pfp-mediated death pathway in the pathogenesis of CIA with the use of pfp-deficient mice (pfp^-/-^) by examining the effect of the mutation on the clinical course of disease, immune response to collagen and on joint pathology.

## Materials and methods

### Mice

Perforin-deficient mice were not available on the DBA/1J background. We obtained pfp^-/- ^C57BL/6J (B6) mice, generated as described previously [15], from the Jackson Laboratories (Bar Harbor, ME, USA). These mice were backcrossed onto the CIA-susceptible DBA/1J background (Harlan-Winkelmann, Borchen, Germany) for at least 14 generations. The mice were propagated as hemizygous mutants and the mutations were followed by PCR analysis of tail DNA. To produce homozygous pfp^-/- ^mice for the experiments, heterozygous pfp-deficient mice were intercrossed. Successful backcrossing to the DBA/1J background was assessed by PCR analysis of the MHC-H2 locus [20]. To select mice heterozygous for the pfp-deficiency the primers 5'-TTT TTG AGA CCC TGT AGA CCC A-3' (pfp1) and 5'-GCA TCG CCT TCT ATC GCC TTC T-3' (pfp2) were used. For selection of homozygous pfp-deficient mice, pfp3 primer (5'-CCG GTC CTG AAC TCC TGG CCA A-3') was used in combination with pfp4 primer (5'-CCC CTG CAC ACA TTA CTG GAA G-3'). Microsatellite markers (Metabion GmbH, Planegg-Martinsried, Germany) surrounding the pfp gene were used for determining the size of the C57BL/6J DNA fragment in the mutant mice. The amplified microsatellites were separated and analyzed on denaturing polyacrylamide gels, and were detected with a LI-COR Model 4200L automated DNA sequencer (LI-COR Inc., Lincoln, NE, USA). For the experiments male mice 8 to 16 weeks old were used. Animals were kept and bred under standard conditions at the facility of the University of Rostock. All experiments were approved by the appropriate authorities in the state of Mecklenburg-Vorpommern, Germany.

### Induction and clinical evaluation of collagen-induced arthritis

Age-matched mice were immunized intradermally at the base of the tail with 125 μg of bovine CII (Chondrex, Redmond, WA, USA) emulsified in CFA (incomplete Freund's adjuvant containing 4 mg/ml *Mycobacterium tuberculosis*; Difco Laboratories, Detroit, IL, USA) or with CFA only. Mice were then boosted with 125 μg of bovine CII in incomplete Freund's adjuvant at day 21. Blood were taken at day 0 and day 21 before boosting, and serum was collected. Clinical scores were assessed immediately before immunization (day 0) and thereafter three times weekly. Inflammation of the four paws was scored as follows: 0, no inflammation; 1, swelling or redness of one joint; 2, swelling or redness of more than one joint or mild inflammation of the whole paw; 3, severe inflammation of whole paw or ankylosis. CFA-immunized mice served as controls.

### T cell proliferation response

Popliteal, preperioteneal, inguinal, mesenterial, axillary and cervical lymph nodes were removed under aseptic conditions. Single-cell suspensions of mononuclear cells of pooled lymph nodes from individual mice were prepared. The cells were cultured in triplicates in flat-bottomed 96-well culture plates, at a concentration of 2 × 10^6 ^cells in 200 μl of medium (RPMI 1640 with Glutamax-II supplemented with 50 IU/ml penicillin, 60 μg/ml streptomycin (Gibco, Karlsruhe, Germany) and 5% heat-inactivated fetal calf serum). To investigate the antigen-specific response, lymphocytes were stimulated with 10, 1 or 0.1 μg/ml bovine CII; 4 μg/ml concanavalin A (Difco) was used as positive control, and medium only was used as negative control. Cells were incubated for 72 hours at 37°C in a humidified atmosphere containing 5% CO_2_. To measure the proliferation by DNA synthesis, cells were pulsed with 1 μCi of [^3^H]thymidine for the last 12 hours of culture. Cells were harvested onto glass fiber filters, and [^3^H]thymidine incorporation was measured in a liquid β-scintillation counter. The results were expressed as counts per minute.

### Cytokine ELISA

To analyze cytokine production, cells were cultured as described above; supernatant was collected after 72 hours *in vitro *antigen challenge. Concentrations of IFN-γ in the supernatants were determined by the Cytoscreen Immunoassay Kit (BioSource, Camarillo, CA, USA) in accordance with the instructions of the manufacturer. In brief, the lymphocyte supernatant was added to an ELISA-plate coated with a monoclonal antibody specific for mouse IFN-γ. After incubation and washing, a biotinylated polyclonal antibody specific for mouse IFN-γ was added. Then streptavidin–peroxidase and later the substrate solution were added to detect the products of the reaction.

### Anti-CII antibody assay

The serum from the mice was analyzed with ELISA for the quantification of IgG antibodies against CII. Micro-ELISA plates were coated overnight at 4°C with 50 μl of PBS containing 5 μg/ml bovine CII in each well. After washing, the sera were added and incubated at 37°C for 2 hours. After 1 hour of incubation at 37°C with anti-mouse-IgG conjugated with alkaline phosphatase (Pharmingen BD), *p*-nitrophenylphosphate containing substrate buffer (Sigma) was added, and 3 M NaOH was used to stop the reaction. The plates were read at 405 nm.

### Histopathology

Mice were killed and paws were cut off and subsequently fixed in 4% paraformaldehyde solution. After decalcification for 2 to 3 weeks in an EDTA solution the paws were embedded in paraffin. The paws were sectioned and stained with H & E. Evaluation of disease was made according to a previously published scale [21]: 1, synovial hyperplasia; 2, start of pannus development; 3, erosions of bone and cartilage; 4, severe inflammation and erosions.

### Fluorescence-activated cell sorting analysis

For the determination of T cell, B cell and NK cell populations in the different pfp-mice, lymphocytes were isolated and stained with fluorescein isothiocyanate (FITC)-labeled anti-CD4 antibody (Pharmingen; clone H129.19), phycoerythrin-labeled anti-CD8a antibody (Pharmingen; clone 43-6.7), FITC-labeled anti-CD45R/B220 antibody (Pharmingen; clone RA3-6B2), phycoerythrin-labeled anti-CD90 antibody (Pharmingen; clone OX-7), and anti-IgG-biotin and streptavidin-FITC before being analyzed by FACScan (Becton Dickinson; with Cell Quest software version 1.2.2).

### Statistics

Statistical evaluation was performed with SPSS software. For analyzing differences in clinical scores, a Mann–Whitney test was used. For incidence calculations, χ^2 ^and Fisher tests were used. When analyzing differences in cytokine production and T cell proliferation, Student's unpaired *t*-test was used. Differences were considered significant at *P *< 0.05.

## Results

### Characterization of the pfp-deficient DBA/1J mice

The pfp-deficient mice were originally derived from C57BL/6J strain by embryonic stem cell transfer. Because this strain is resistant to CIA induction, we backcrossed the mice for at least 14 generations into the CIA-susceptible DBA/1J background. The pfp gene is located on chromosome 10 at 36 cM, and the mutant gene was inherited as a C57BL/6J fragment of about 10 cM between about 28 and 39 cM (Table 1). To exclude the possibility that backcrossing with the DBA/1J strain revealed a defect in T cell, B cell or NK cell maturation we examined distribution of these different immune cell populations by fluorescence-activated cell sorting. It was found that T lymphocytes expressing CD4 and CD8, as well as CD90^+ ^cells (CD90 is expressed on T cells and NK cells) and CD45R/B220-positive cells (B cells, activated killer cells) were present at comparable percentages in heterozygous and pfp-deficient DBA/1J mice, indicating that the lack of pfp did not affect development of these cell populations in the DBA/1J strain (data not shown). The lack of pfp expression in the activated T cells of the pfp^-/- ^mice was confirmed at the RNA level by RT-PCR (see Additional file 1a).

### Pfp-deficient mice are less susceptible to collagen-induced arthritis

**Figure 1:**
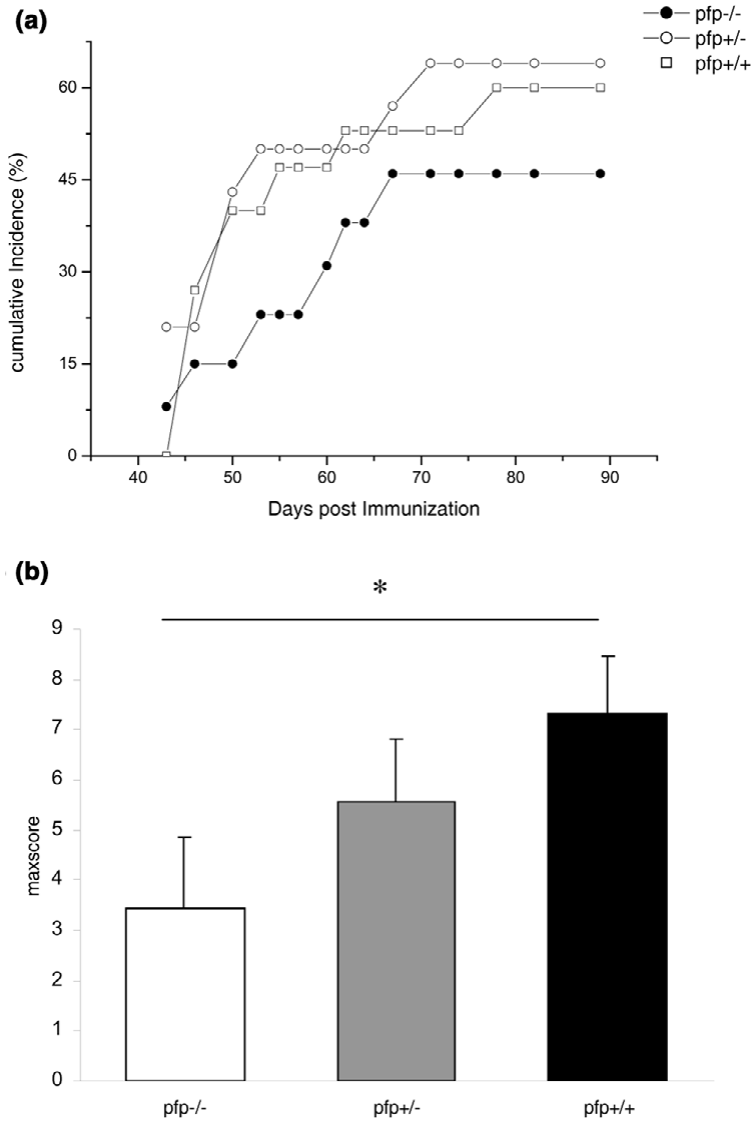
Perforin-deficient (pfp^-/-^) mice develop decreased collagen-induced arthritis (CIA). Percentage of CIA-affected animals **(a) **is decreased and average maximum score **(b) **is significantly lower in pfp^-/- ^mice than in pfp^+/- ^and pfp^+/+ ^mice. Figures show results from two different experiments, with balanced groups, taken together. Maxscore was calculated as the mean maximum score for each individual mouse of a given genotype. *P < 0.05.

To investigate the role of pfp in collagen-induced arthritis, we immunized DBA-pfp^-/-^, DBA-pfp^+/- ^and DBA-pfp^+/+ ^mice with bovine CII in CFA. As shown in Fig. 1 and Table 2, mice deficient for pfp developed a less severe disease with lower clinical scores than mice with intact pfp. DBA-pfp^-/- ^mice also showed a tendency to a delayed onset of CIA. Mice with intact pfp (pfp^+/+ ^and pfp^+/-^) had an average incidence of 64%, whereas pfp^-/- ^mice showed a mean incidence of 54% (Fig. 1). The severity of disease was decreased on day 50 after immunization (pfp^-/-^, 0.9 ± 0.86; pfp^+/-^, 1.4 ± 0.67; pfp^+/+^, 1.3 ± 0.47 (means ± SEM)), and on day 75 (pfp^-/-^, 2.1 ± 1.7; pfp^+/-^, 3.8 ± 1; pfp^+/+^, 5.2 ± 1.2), and significantly decreased on day 82 (pfp^-/-^, 3.0 ± 1.5; pfp^+/-^, 4.0 ± 0.9; pfp^+/+^, 5.6 ± 0.9; *P *< 0.05). Maxscore, calculated as the mean of the maximum score value for each individual mouse of a given genotype, was also significantly decreased in pfp-deficient mice (pfp^-/- ^3.4 ± 1.45) in comparison with pfp^+/+ ^mice (7.3 ± 1.14; Fig. 1). In addition, the area under the curve, as a measure of severity, onset and chronicity, was significantly lower in pfp^-/- ^mice than in mice carrying pfp (pfp^-/-^, 31.9 ± 23; pfp^+/-^, 44.6 ± 12.3).

### Histopathological features of CIA do not change with pfp deficiency

**Figure 2:**
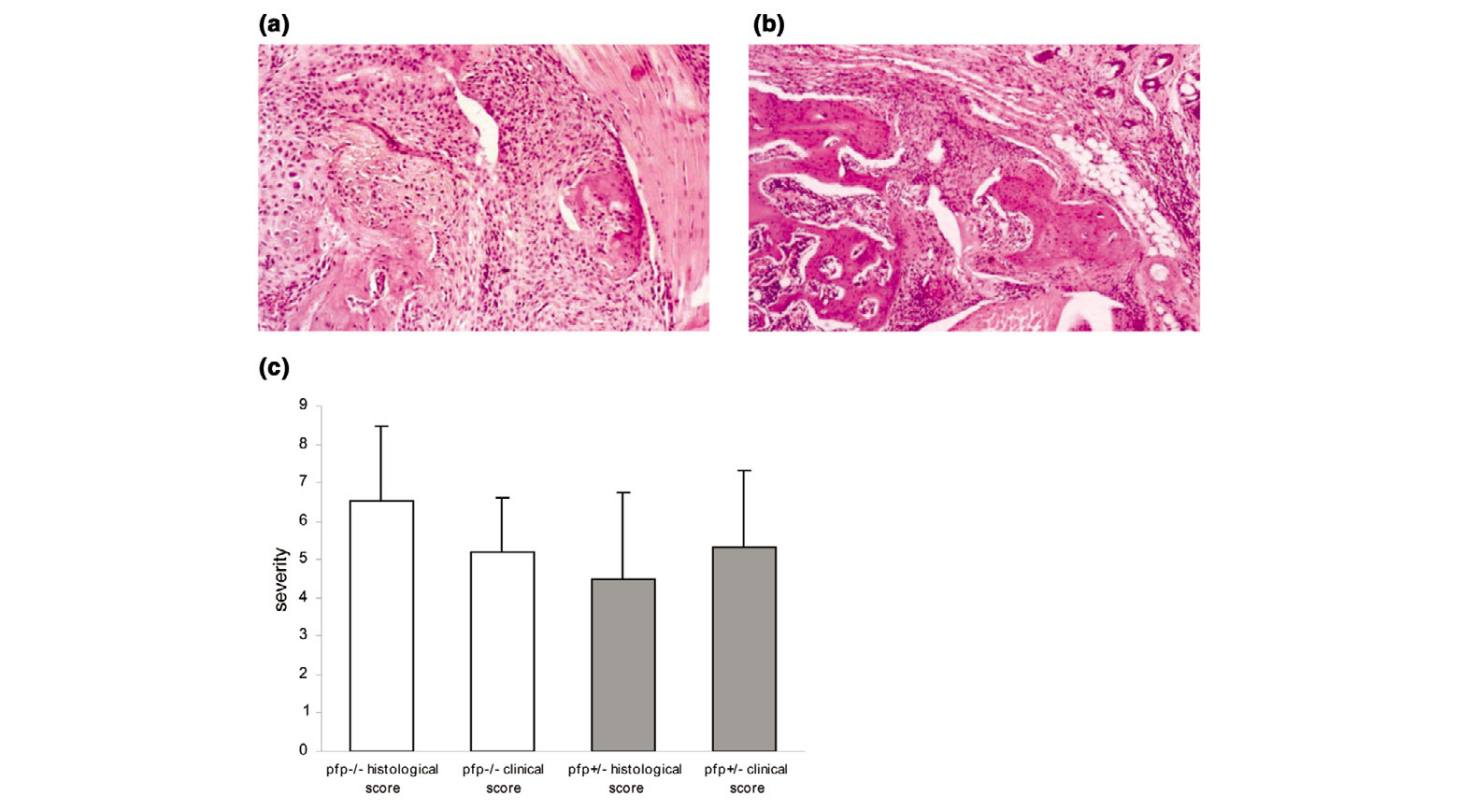
Histopathology of perforin-deficient pfp^-/- ^mice compared with pfp^+/- ^mice. **(a) **Joint of a pfp^-/- ^mouse with severe inflammation and erosions, stage 4. **(b) **Paw of a heterozygous mouse in the same stage of disease. **(c) **Comparison of histological and clinical evaluation of the disease. Data are values of severity of pfp^+/- ^mice (*n *= 6) and pfp^-/- ^mice (*n *= 6), and are shown as means ± SEM.

To investigate joint histopathology, paws were dissected from pfp^-/- ^and pfp^+/- ^mice with CIA, H & E-stained and evaluated blind for signs of arthritis. Results (Fig. 2) reveal that pfp^-/- ^mice can develop a severe arthritis with typical signs of CIA, namely proliferation of synoviocytes, infiltration of inflammatory cells, pannus development, and erosions of bone and cartilage. Comparing both genotypes, no differences in histopathological development of the disease were visible (Fig. 2).

### Lack of pfp does not affect the antibody response to collagen

**Figure 3:**
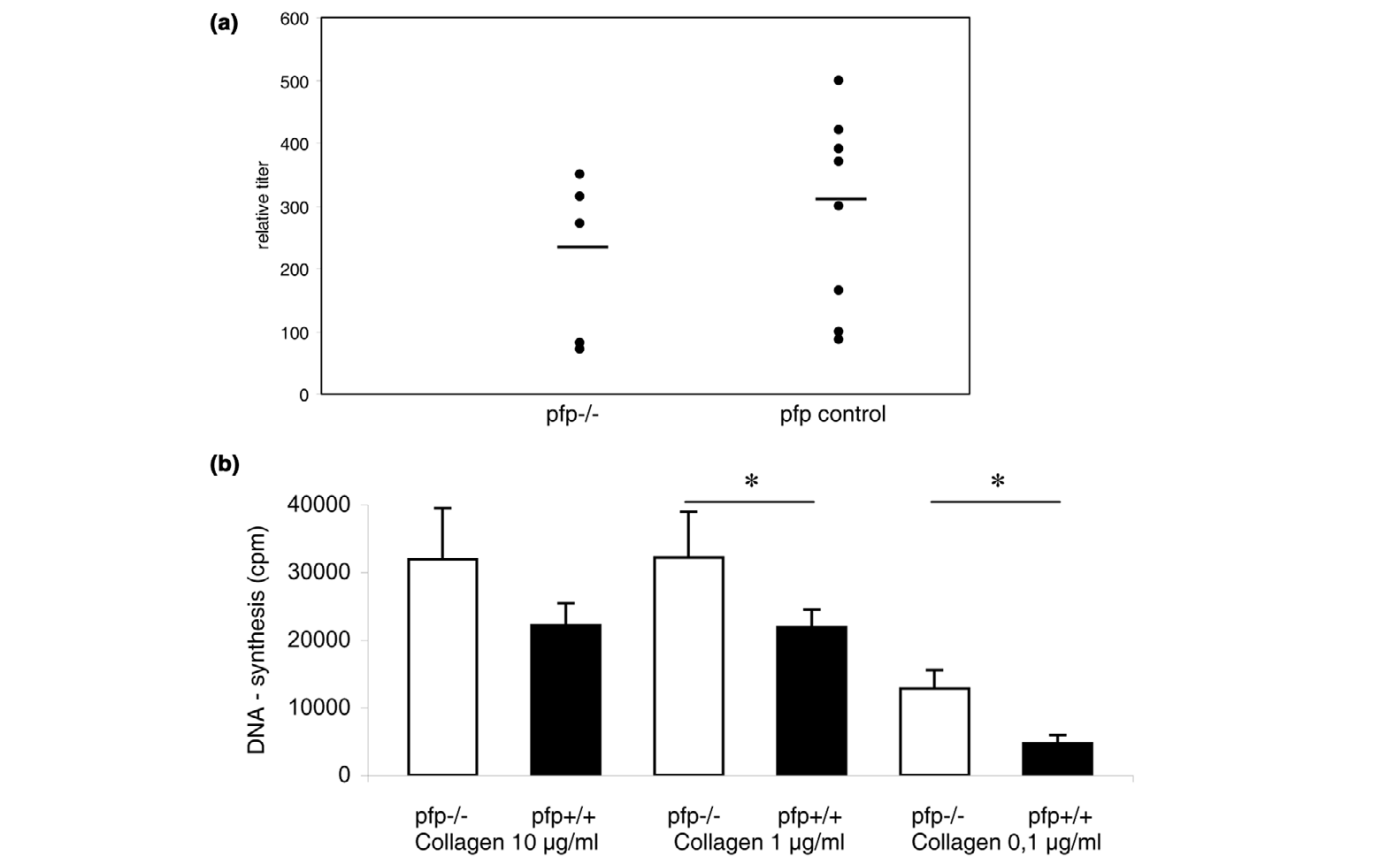
Antibody response and T cell proliferation. **(a) **No significant variations in the levels of collagen-specific IgG antibodies between the perforin (pfp)-deficient mice (*n *= 6) and the control littermates (pfp^+/- ^and pfp^+/+^; *n *= 10) were seen. Sera were obtained from the mice 21 days after immunization with collagen type II (CII) and complete Freund's adjuvant (CFA). Levels of collagen-specific IgG antibodies were measured by quantitative ELISA. **(b) **pfp^-/- ^mice show an increased proliferation toward CII. Draining lymph nodes were obtained from pfp^-/- ^(*n *= 3) and pfp^+/+ ^(*n *= 3) mice 90 days after immunization with CII in CFA. The cells were cultured for 60 hours with collagen II in different concentrations, and then pulsed with [^3^H]thymidine. *P < 0.05.

To investigate the role of pfp on the humoral immune response to collagen, sera were obtained from pfp^-/-^, pfp^+/- ^and pfp^+/+ ^mice at days 0 and 21 after immunization, and the levels of CII-specific IgG antibodies were measured by quantitative ELISA. No significant variations were seen in the levels of anti-CII-specific IgG between the pfp^-/- ^and pfp^+/- ^or pfp^+/+ ^littermates (Fig. 3). Pfp-deficient mice showed a mean titer of anti-CII antibodies of 233 ± 51.1, and the control group had a mean titer of anti-CII antibodies of 306 ± 50.8 at day 21 after immunization. These data show that even without pfp the mice develop a strong humoral immune response against CII, suggesting that the lack of pfp does not affect the anti-CII antibody response.

### Pfp^-/- ^mice mount an enhanced proliferative T cell response toward CII

To investigate whether the T cell response against CII was affected by pfp deficiency, draining the lymph nodes of immunized pfp^-/- ^and pfp^+/+ ^was investigated for the proliferative response toward bovine CII. Perforin-deficient mice showed an enhanced T cell proliferation compared with pfp wild-type mice. As shown in Fig. 3, after stimulation with 0.1 μg/ml CII, pfp^-/- ^mice showed a significantly elevated T cell proliferation of 12,835 ± 2,750 cpm compared with 4,727 ± 1,268 cpm in pfp^+/+ ^mice; at a CII concentration of 1 μg/ml the T cell proliferation was also significantly increased in pfp-deficient mice (32,209 ± 6,764 cpm) compared with pfp-wild-type mice (21,904 ± 2,626 cpm). The IFN-γ production of T cells from these mice was measured by ELISA, but no significant differences between pfp^-/- ^and pfp^+/+ ^mice were observed (data not shown).

## Discussion

The primary aim of this study was to investigate whether the delayed onset and mild arthritis observed earlier in CD8^-/- ^mice [19] is due to a lack of cytotoxic ability or a lack of some other aspects of T cell function such as the secretion of cytokines. Indeed, we found that pfp-deficient mice develop disease with reduced incidence, a slightly delayed onset, and significantly decreased severity, suggesting that CTL activity is important for the initiation and maintenance of arthritis in the CIA model. This is in agreement with earlier observations in the insulin-dependent diabetes mellitus (NOD) mouse model. In that model, as in CIA, pfp is a susceptible rather than a protective gene. This is in contrast to other disease models, for example experimental autoimmune encephalomyelitis (EAE), systemic lupus erythematosus (SLE) and autoimmune pancreatitis, in which pfp clearly protects against the disease [16-18,22]. The protective effects of pfp could be explained by its immune regulatory function, namely the killing of autoreactive B cells and functional self-lysis of activated T cells through AICD. Hence, its role in regulation of peripheral tolerance and its role in autoimmunity, which is distinct from, but overlaps, the role of the FasL/Fas and TNF/TNFR pathways, might be the reason for accelerated autoimmunity in pfp-deficient mice in the EAE and SLE models.

In the present study we obtained evidence that pfp can also act as a susceptibility gene rather than a protective gene. A lack of the molecule led to reduced severity throughout the disease, a slightly delayed onset, and a reduced incidence of CIA. However, we also observed a strong anti-collagen B cell response with similar anti-collagen-specific IgG levels in control and pfp^-/- ^mice, and significantly increased T cell proliferation in response to collagen. This might have been due to a reduced killing and accumulation of autoreactive T cells after activation because of impaired AICD. These findings indicate that a diminished adaptive response to collagen was not responsible for the reduced arthritis.

Previous studies demonstrated a strong involvement of pfp in the control of CD8^+ ^T cell homeostasis. Perforin deficiency resulted in enhanced CD8^+ ^T cell expansion because of decreased killing of antigen-presenting cells and consequential prolonged stimulation by antigen [23,24]. Our results support these propositions: we observed a significantly increased T cell proliferation in pfp-deficient mice in comparison with controls. These data also suggest a role of pfp in the regulation of T cell homeostasis. Nevertheless, cytokines produced by cytotoxic T cells are also involved in the regulation of the T cell response. TNF-α, for example, can mediate AICD of CD8^+ ^T cells through TNFR1 and TNFR2 [25].

The histopathology of CIA in wild-type pfp^+/+ ^mice of the present study was similar to that seen in previous studies [26]. The data are therefore not shown again here. In the present study there were no significant differences between histopathological changes of pfp^+/- ^and pfp^-/- ^mice, most probably because of a severe disease in individual pfp^-/- ^mice. This result argues for an additional involvement of pfp-independent death pathways in joint destruction. Indeed, pfp was upregulated in the inflamed arthritic joints of wild-type mice as shown by RT-PCR (Additional file 1b).

This is similar to our recent finding showing that the FasL/Fas pathway has a proinflammatory role in CIA and an activating function on fibroblasts *in vitro *[27]. Fas-deficient mice developed arthritis that was less severe, probably through a reduced IL-1R1/Toll-like receptor-4 signaling that might contribute to a decreased expression of other cytokines, chemokines and matrix metalloproteinases potentially regulated by this pathway [28]. Previous studies also reported a strong involvement of the TNF/TNFR pathway because CIA only developed with a reduced disease incidence, and the severity and neutralization of TNF led to the prevention of arthritis [29,30].

Taken together, the results show that in CIA the disease-promoting effect of pfp prevails. It is therefore tempting to speculate that pfp could contribute to arthritis in at least two ways. First, it could promote tissue damage by direct cytotoxic effects through CD8^+ ^T cells and NK cells. Second, pfp might have some activating functions on fibroblasts or macrophages, leading to the production of proinflammatory cytokines. Indeed, there are reports indicating that fibroblasts and monocytes can be activated by granzyme A to secrete the proinflammatory cytokines IL-6, IL-8 and TNF-α, which could subsequently severely regulate the inflammatory response [31,32].

There are other indications that argue for a prominent role of pfp in arthritis. Perforin was found to be differentially expressed in lymph nodes and joints of DBA/1J and FVB/N (CIA-resistant strain) mice (SM Ibrahim and D Koczan, unpublished observations) and pfp-expressing cytotoxic T lymphocytes and increased apoptosis were observed in the synovia of patients with rheumatoid arthritis [10,33,34]. The targeted pfp gene is likely to have a major role in the observed effects, on the basis of the absence of pfp production, although it is possible that other polymorphic genes in the linked region could also contribute to CIA reduction. Indeed, the pfp gene is mapped to a CIA-susceptible locus, *Cia8 *on chromosome 10. This quantitative trait locus is covered by the 12 cM C57BL/6J fragment including the mutant pfp gene. The *Cia8 *region contains several candidate genes that have been implicated in the modulation of CIA susceptibility, for example the macrophage migration inhibitory factor Mif [35] and the autoimmune regulator Aire [36]. However, the observation that heterozygous littermates and pfp^+/+ ^mice that were also backcrossed and contained the same or smaller C57BL/6J fragments do not show effects on the disease argues against a major role for another gene.

In summary, the CIA in pfp-deficient mice was mild and showed delayed onset and reduced incidence, but some individual pfp^-/- ^mice also developed a severe disease. These results suggest that pfp-dependent cytotoxicity is involved in the initiation of tissue damage in arthritis, but that pfp-independent cytotoxic death pathways, for example the FasL/Fas pathway, might also contribute to CIA.

## Conclusion

We report that arthritis developed only with reduced incidence, severity and delayed onset in pfp-deficient DBA/1J mice. These findings suggest that pfp-dependent cytotoxicity is involved in the initiation of tissue damage in arthritis but also that one or several other pfp-independent mechanisms, possibly involving FasL/Fas, contribute to the early phase of joint destruction in CIA.

## Abbreviations

AICD = activation-induced cell death; CFA = complete Freund's adjuvant; CIA = collagen-induced arthritis; CII = collagen type II; CTL = cytotoxic T lymphocyte; EAE = experimental autoimmune encephalomyelitis; ELISA = enzyme-linked immunosorbent assay; FITC = fluorescein isothiocyanate; H & E = hematoxylin and eosin; IFN = interferon; IL = interleukin; MHC = major histocompatibility complex; NK = natural killer; RT-PCR = reverse transcriptase polymerase chain reaction; pfp = perforin; SLE = systemic lupus erythematosus; TNF = tumor necrosis factor.

## Competing interests

The author(s) declare that they have no competing interests.

## Authors' contributions

KB, AK and HTR did the experimental work and contributed to writing the manuscript. KB and AK contributed equally to the work. DK, HJT, RH and SMI participated in the design and co-ordination of the study and drafted the manuscript. HJK and HN did the histopathology. EM carried out the T cell proliferation and cytokine assays. All authors read and approved the final manuscript.

## Supplementary Material

Additional File 1 A TIFF file showing the perforin mRNA expression level in CD8+ T cells of perforin-deficient, heterozygous and wild-type mice as well as the perforin mRNA expression level of healthy and inflamed joints from wild-type mice. Click here for file

## Figures and Tables

**Table 1:** Mapping of DBA perforin-deficient mice

Marker	Position (cM)	Parental
D10Mit38	26.8	DBA
D10Mit44	27	DBA
D10Mit194	29	C57BL/6J
D10Mit61	32	C57BL/6J
D10Mit20	35	C57BL/6J
D10Mit31	36	C57BL/6J
D10Mit32	38	C57BL/6J
D10Mit186	40	DBA
D10Mit174	41	DBA
D10Mit175	41.8	DBA
D10Mit42	44	DBA
D10Mit261	47	DBA
D10Mit67	49	DBA

The mice were backcrossed for 14 generations to the DBA/1J background.

**Table 2:** Summary of disease-related data of perforin-deficient (pfp^-/-^) mice compared with pfp^+/- ^and pfp^+/+ ^mice

Genotype (*n*)	Day of onset	Incidence (%)	Severity			Maxscore	AUC
					
			Day 50	Day 75	Day 82		
Pfp^-/- ^(13)	59 ± 4.9	54	0.9 ± 0.8	2.1 ± 1.6	3.0 ± 1.5^a^	3.4 ± 1.4^b^	31.9 ± 23.0^c^
Pfp^+/- ^(14)	52 ± 3.5	64	1.4 ± 0.6	3.8 ± 0.9	4.0 ± 0.9^a^	5.6 ± 1.2^b^	44.6 ± 12.3^c^
Pfp^+/+ ^(14)	53 ± 3.6	64	1.3 ± 0.4	5.2 ± 1.2	5.6 ± 0.9^a^	7.3 ± 1.1^b^	59.4 ± 16.7

Evaluations and analyses were performed as described in Materials and methods. Maxscore was calculated as the mean of the maximum score value for each individual mouse of a given genotype. Data are group mean values ± SEM. Values with the same superscript letter are significantly different (*P *< 0.05). AUC, area under the curve.
