# Supplementary figures and images for: Perforin deficiency attenuates collagen-induced arthritis
Source: Arthritis Res Ther. 2005 May 20;7(4):R877–84. doi: 10.1186/ar1758 (PMC1175039; doi:10.1186/ar1758)

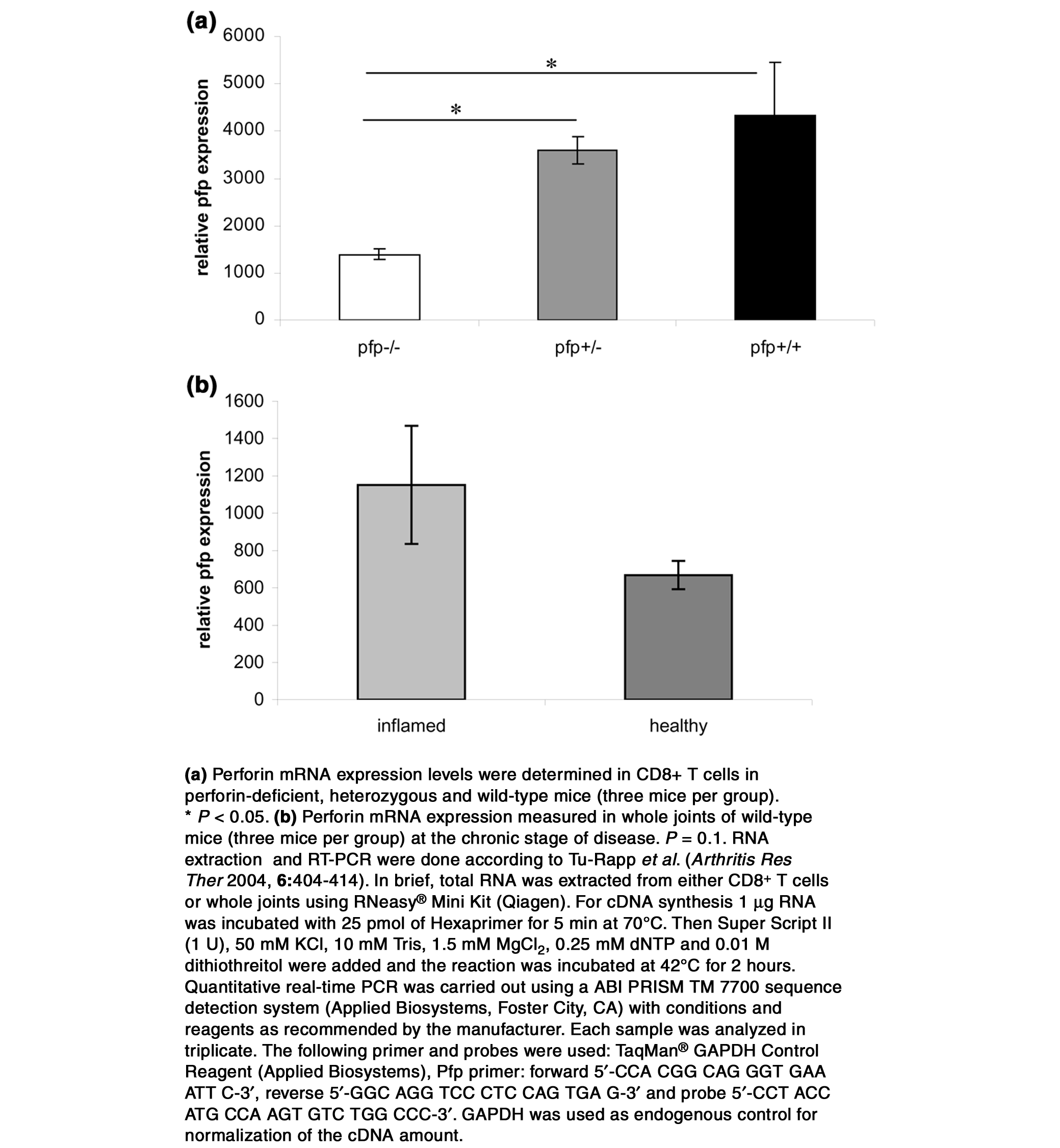

Supplement: Additional File 1 — A TIFF file showing the perforin mRNA expression level in CD8+ T cells of perforin-deficient, heterozygous and wild-type mice as well as the perforin mRNA expression level of healthy and inflamed joints from wild-type mice. [file ar1758-S1.tiff]
